# Does the implementation of a new payment system for hospital services induce changes in the quality of health care? Experiences from Germany

**DOI:** 10.1186/1472-6963-14-S2-O18

**Published:** 2014-07-07

**Authors:** Bertram Haeussler, Karsten Zich, Hans-Holger Bless

**Affiliations:** 1 IGES Institut, Berlin, Germany

## Background

In 2003 Germany started to replace its per-day hospital payment system by a prospective payment system based on diagnosis related groups (DRG). The primary objective of the program was to increase economic efficiency of hospital care. The introduction was accompanied by fears that quality of care could deteriorate when hospitals withhold necessary care in order to increase profits. Outpatient physicians expected an increase in their workload, which could probably lead to financial losses if it were not followed by an increase of their reimbursement. In order to detect possible adverse effects, the Statutory Health Insurance (SHI) was obligated by law to commission independent observational research on this question.

## Materials and methods

There was access to a wide range of information like hospital claims, nationwide quality reporting information; claims from sickness funds. Various surveys of stakeholders were carried out. Observation period (OP) was from 2004 to 2010. The basic approach was the over-time comparison of parameters that were interpreted as indicators for resource use, access to care or quality of care. Possible changes of these indicators over the observation period were reviewed qualitatively if they could have been induced by the change of the payment system.

## Results

Length of stay (LOS) as an indicator for resource use decreased significantly over the OP from 7.8 to 6.8. However, the annual decrease of 2.2% did not significantly differ from the period 1995 to 2004. Average distance between patients’ home and the locations of hospitals (as indicator for access) increased slightly from 22.4 km (31.8 min) to 22.6 km (32.3 min). Post discharge mortality as an indicator for quality of care decreased by 7.8% (30 days after discharge) or 6.5% (90 and 365 days).

## Conclusions

There was little evidence that access changed during the observation period. Resource use, as measured by LOS, was reduced but this trend had already started more than ten years before the program started. This did not support the hypothesis that the change of the payment system was the major cause. Quality as measured by mortality improved over the OP but this could not be attributed to the program. Overall there was no evidence that the program did affect patients negatively.

